# Enhanced protein synthesis and secretion using a rational signal-peptide library approach as a tailored tool

**DOI:** 10.1186/1753-6561-5-S8-O13

**Published:** 2011-11-22

**Authors:** Beate Stern, Asta Optun, Melanie Liesenfeld, Christine Gey, Markus Gräfe, Ian F Pryme

**Affiliations:** 1 UniTargetingResearch AS, Bergen, Norway; 2 Department of Biomedicine, University of Bergen, Norway; 3 Department of Physics and Technology, University of Bergen, Norway

## Background

There is a steadily increasing demand for producing higher yields of biopharmaceuticals as recombinant proteins, and it is anticipated that this will further expand during the next decades. Among the most frequent proteins produced are growth factors, monoclonal antibodies, hormones and blood coagulation factors.

The production of recombinant proteins can be performed in expression systems derived from bacteria, yeast, plants, insects or mammals. Prokaryotic cells divide rapidly, making it possible to produce high yields of the protein at low costs. They are, however, normally not able to perform post-translational modification of proteins, which, for those of mammalian origin, is essential to ensure stability, proper folding and assembly and thus biological activity. Mammalian cells, on the other hand, confer post-translational modification. Unfortunately their cultivation is cost expensive and time consuming, they grow slower, are more sensitive to contamination and produce lower protein yields than their prokaryotic counterparts. Despite this, 60-70% of all recombinant proteins used in therapeutics are produced in mammalian cells. Yield improvement in mammalian systems is currently an area of major industrial importance [1].

To achieve this, two main strategies have been used: (1) optimising the components of the vector containing the gene of interest, and (2) optimising cell growth and selection. Optimisation of the vector by genetic engineering has mainly focused on increasing the efficiency by which the gene is transcribed. The concept being that a high level of transcription would ultimately lead to a higher protein yield due to increased availability of mRNA for translation. Vector design, the chromosomal environment of the plasmid integrated in the host genome and plasmid copy number, are among the parameters that can contribute to transcription efficiency. An increased level of mRNA coding for any secreted protein of interest, however, will only be beneficial if the transcript is correctly transported to the endoplasmic reticulum and then effectively translated. This area has hitherto been largely neglected.

## Efficient mRNA processing

UniTargetingResearch AS is now developing and commercialising tools to optimise protein synthesis and secretion, by ensuring that the mRNA encoding the protein of interest is efficiently processed. This has proved to be heavily dependent on the presence of specific genetic targeting elements, namely a selected signal sequence (SS) in combination with appropriate 5' and 3' untranslated regions (5' and 3'UTRs). Our focus is currently on the SS, which is translated into the signal peptide (SP).

Earlier we observed a competition between a selected SS and the 3'UTR in mediating mRNA targeting to distinct classes of polysomes [2]. We therefore investigated the effect of different SPs derived from mammalian secretory proteins on the synthesis/secretion of *Gaussia princeps* (a marine copepod) luciferase (Gluc) used as a reporter protein [3]. The results showed that the choice of SP had a major impact on synthesis/secretion of Gluc in CHO cells. Contrary to what was expected the SP of albumin was extremely inefficient (<5%) in Gluc production when compared to that obtained using the SP derived from the marine organism. Similar results were obtained when liver cells were used instead of CHO cells, such that the effect was not cell specific. Other SPs derived from mammalian proteins (chymotrypsinogen, trypsinogen-2 and interleukin-2) also resulted in much lower yields of Gluc. Since in all cases the levels of mRNA coding for Gluc were relatively the same, the importance of a post-transcriptional event was strongly indicated.

Further studies were performed on a series of other marine SPs, including that from Oikosin 1 (Oik1), a protein from *Oikopleura dioica*, and mutants thereof. Twenty constructs of such a mutant pool generated by randomising the codons encoding the amino acids (AAs) in positions 4, 5 and/or 13 were randomly selected and tested for synthesis/secretion of Gluc (Table 1). Large differences in yield were observed, ranging from 0 (mutant 20) to almost 250% (mutant 1). Eleven of the mutants resulted in lower levels of luciferase yield than the wild-type, while 9 showed higher levels. The results thus demonstrated that a high yield was extremely dependent on the AA sequence and even a single mutation at a specific position has a strong effect on protein yield. In order to test whether or not the total hydrophobicity of the hydrophobic core region (h-region) in the SP was of importance, hydropathy scores according to Eisenberg *et al. *[4] were calculated. From Table 1 it can be seen that there appears to be no direct correlation between yield and degree of hydrophobicity of the h-region and thus the latter is not a reliable measure for the prediction of the efficiency of an individual SP.

**Table 1:** Levels of luciferase yield in the medium of CHO cells transfected with plasmids encoding Oik1 SP wild-type and 20 Oik1 SP mutants randomised in positions 4, 5 and/or 13 (mutated residues underlined), and hydropathy scores of the AAs in the respective SPs.

SP	AA sequence	Protein yield in medium^a)^	Hydropathy score^b)^
Wild-type	MLLLSALLLGLAHGYS	100	100
Mutant 1	MLLLSALLLGLAFGYS	246	139
Mutant 2	MLLLSALLLGLACGYS	224	117
Mutant 3	MLLSFALLLGLALGYS	222	139
Mutant 4	MLLVLALLLGLALGYS	201	167
Mutant 5	MLLLSALLLGLAMGYS	191	126
Mutant 6	MLLRIALLLGLAHGYS	188	49
Mutant 7	MLLLSALLLGLAIGYS	185	144
Mutant 8	MLLLPALLLGLAHGYS	142	107
Mutant 9	MLLVPALLLGLAHGYS	126	108
Mutant 10	MLLQYALLLGLAHGYS	89	64
Mutant 11	MLLLSALLLGLAPGYS	89	113
Mutant 12	MLLLSALLLGLARGYS	82	47
Mutant 13	MLLKVALLLGLAAGYS	77	94
Mutant 14	MLLRTALLLGLAWGYS	74	43
Mutant 15	MLLKVALLLGLAHGYS	70	69
Mutant 16	MLLLSALLLGLAAGYS	70	125
Mutant 17	MLLFSALLLGLAHGYS	63	103
Mutant 18	MLLVQALLLGLAHGYS	63	84
Mutant 19	MLLRGALLLGLAVGYS	12	64
Mutant 20	MLLEHALLLGLAHGYS	0	50

^a)^ Average protein yield obtained from 3 independent transient transfections, given in percent with respect to the value obtained with Oik1 wild-type set to 100%.

^b)^ Sum of hydropathy scores of AAs at positions 4-13, judged to comprise the h-region of the Oik1 SP/SP mutants, given in percent with respect to the score for Oik1 wild-type set to 100%.

## The library concept

**Figure 1:**
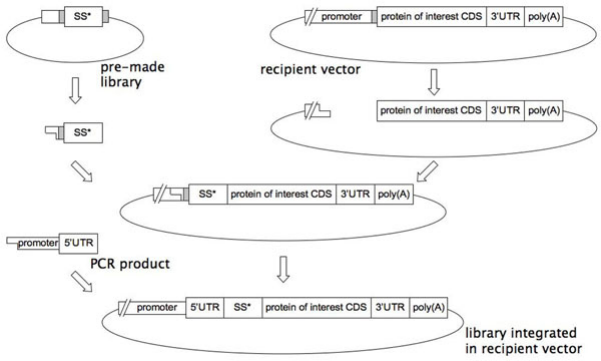
Seamless insertion of a randomised SS (SS*) from a pre-made library into a recipient vector. Grey boxes indicate special restriction sites used in the cloning process.

We thus adopted an alternative approach where we exploit the plethora of biological data we have accumulated in a bioinformatics context. A comparison of the success of individual SPs and an analysis of their AA composition has allowed us make predictions with respect to which AAs in which positions are likely to have a decisive influence on protein synthesis/secretion. Based on this we have developed a tool, termed UTR^®^Tailortech, that provides us with the opportunity of generating rational SP libraries randomised at chosen positions. In contrast to a traditional non-rational approach which results in libraries of astronomic proportions not being manageable, with UTR^®^Tailortech the libraries are considerably reduced in size while simultaneously being enriched for good performers. This increases the probability of finding “the needle in the haystack”. The tool also comprises the concept of generating libraries that are re-usable by constructing so-called “pre-made” libraries. These high-quality libraries can be linked in a seamless manner to any protein-coding region contained in any expression vector as outlined in Fig. 1. When combined with high-throughput screening technology, a tailored SP for any specific protein (including difficult-to-express proteins) can readily be defined.

## Conclusion

From our studies it is evident that there is considerable room for improvement of production of a recombinant protein by genetically modifying the SP utilised in a vector. To our knowledge UTR^®^Tailortech is the first attempt to harness this potential in a rational way.
